# Primum Non Nocere

**DOI:** 10.21315/mjms2021.28.1.17

**Published:** 2021-02-24

**Authors:** Hans Van Rostenberghe

**Affiliations:** Department of Paediatrics, School of Medical Sciences, Universiti Sains Malaysia, Kelantan, Malaysia

**Keywords:** mRNA, vaccine, COVID-19, safety, side effects

## Abstract

The coronavirus disease 2019 (COVID-19) pandemic is severe and has not shown any signs of warning up to today. Biotech companies around the world have raced to come up with an acceptable vaccine and recently two mRNA vaccines have received emergency usage authorisation from regulatory bodies in several countries. mRNA vaccines, which consist of a new and revolutionary technology have not been previously tested widely on humans. Medium- and long-term safety data are not available. While many experts seem to support the start of a mass vaccination campaign, others feel there are too many unknowns to embark on a mass vaccination campaign. Concerns include uncertainties about the long-term effects of foreign mRNA on human cellular physiology and the possibility of vaccine-enhanced disease severity, which may not be unlikely with the current disease presentation of COVID-19.

Dear Editor,

## Introduction

The coronavirus disease 2019 (COVID-19) pandemic has raged throughout 2020 and has until now not abated. Up to mid-January 2021, the pandemic has caused more than two million confirmed deaths worldwide (1) and many nations have faced a critical situation with insufficient intensive care unit (ICU) beds for severely affected patients. No one in his right mind can deny the severity of the pandemic.

Biotech companies around the world entered a race to come up with effective and safe vaccines. Less than 10 months ago, the first vaccine trial started only weeks after the World Health Organization (WHO) declared the COVID-19 a pandemic. The two vaccines that seemed to move fastest were both messenger ribonucleic acid (mRNA)-based vaccines, which had not previously been widely tested on humans. On 11 December 2020, under strong political pressure, the Food and Drug Administration (FDA) in the United States granted an emergency use authorisation for the first of these mRNA-based vaccines. The second mRNA vaccine received similar authorisation on 20 December 2020.

## The Trial of the First Approved Vaccine (2)

Trial participants self-reported the adverse events, and headache and fatigue were reported in more than 50% of the vaccine recipients versus in around 20% of placebo recipients (2). “The pharmaceutical company was responsible for the design and conduct of the trial, data collection, data analysis, data interpretation and the writing of the manuscript” (2). The writers of the manuscript interpret the vaccine to be safe and efficacious (2). It is highly recommended for anyone involved in vaccine delivery to read and critically appraise the full reports of the trials.

On a leisurely or superficial reading of the report, it seems easy to agree with the writers of the manuscript (2). But it only takes a few questions beyond that to become aware of some significant concerns about long-term safety. The questions below are merely questions and are not meant to suggest that there is evidence in one or the other direction. We simply do not have the data.

## Potential Concerns

The mRNA will enter our cells and our ribosomes will produce the viral protein. Do we know at this time which cells of our body are participating in the production of the protein and what happens to the cells that participate in the process? The vaccines are designed to target the dendritic cells. But are other cells involved too? Could the glial cells be involved? Or the muscle cells? Or even the myocytes of our heart? How about other cells? Headache and lethargy were among the two main side effects of the vaccine. Are the cells producing the protein attacked by our immune system and annihilated? Are there long-term effects of a potential loss of these cells?

The regulation of our gene expression (why some cells express certain genes and other cells express other genes) by individual cells is far from completely understood today. Is the ribosomal production of protein in our cells in any way having an effect or feedback on the regulation of gene expression?

Then, there is the question of vaccine enhancement of disease severity, also called antibody-dependent enhancement (ADE). Even though the above-mentioned manuscript (2) mentions an unjustified optimistic statement about this issue, this may be among the biggest long-term concerns, especially if patients in the future get infected with mutated strains of the virus. It took another company close to 10 years to research the safety of Dengvaxia, a dengue vaccine, but it took only a very short time after mass vaccination for disaster to strike in the Philippines, with children experiencing increased disease severity after receiving Dengvaxia (3). Vaccine-associated enhanced disease was first described more than 50 years ago, when a vaccine against respiratory syncytial virus (RSV) was tested. When infected by RSV, 80% of vaccinated children required hospitalisation (and some died), while only 5% of virus-exposed controls needed hospitalisation (4).

In a very recent report, Norway reported 23 deaths among the elderly within a very short time after they received the first approved mRNA COVID-19 vaccine, raising some questions about short-term safety for certain groups (5).

## Interpretation

Proponents of the mRNA vaccines state that based on what we know now (which is extremely far from everything) about cell physiology and our immune system, long-term negative effects of the newly designed mRNA vaccines are unlikely and that the ratio of risk to expected benefit may be small. The author holds the view that awaiting the full results of research on the non-mRNA vaccines that are being developed against COVID-19 may be a safer option for mass vaccination. These vaccines resemble, at least a bit, the classical vaccines that have a proven safety profile. The risk for ADE, however, may not be different from the mRNA vaccines, and their reported efficacy may be lower than that of the mRNA vaccines (6).

Vaccines are among the preventive strategies administered to healthy individuals who are at risk of certain infections. Mass vaccination has been proven to be useful, safe and effective in the eradication of smallpox, the control of polio and measles and many other diseases. Vaccines, currently included in the (Malaysian) schedule for children, are highly recommended based on their proven efficacy and short- and long-term safety, which was demonstrated in the extensive development phase that took years in each case. The author of this letter supports 100% vaccines that are currently included in the vaccination schedule for Malaysian children and he disagrees completely with ‘antivax’ parents who refuse these proven effective and safe vaccines for their children.

Currently, however, we are confronted with vaccines against COVID-19, some of which use a completely new and revolutionary technology. The seriousness of the pandemic has led to the regulatory emergency use authorisation of these vaccines in several countries under strong political pressure. Performing preventive mass immunisation campaigns on healthy individuals required very solid safety data in the past. The long-term safety of the COVID-19 vaccines is at this moment uncertain. Their medium-term safety is also uncertain.

We all hope everything will go right. However, is mere hope, without solid data, enough? If something goes wrong in the long term, who will take responsibility? The regulatory bodies (not granting approval but giving emergency usage authorisation)? The big companies producing the vaccines? Or does the responsibility lie with us, the prescribing doctors? None of the above-mentioned groups are likely to be held responsible in human courts. However, when we graduated as medical doctors, we all took a solemn oath: **primum non nocere** (first, do no harm). Whether to recommend and administer a new vaccine with an uncertain long-term safety profile on a massive number of healthy persons poses a challenge to the consciousness of the doctors who dare to consider some questions beyond the obvious and the superficial.
